# The investigation of Mitogen-Activated Protein kinase Phosphatase-1 as a potential pharmacological target in non-small cell lung carcinomas, assisted by non-invasive molecular imaging

**DOI:** 10.1186/1471-2407-10-95

**Published:** 2010-03-12

**Authors:** Cheng-Jeng Tai, Alexander TH Wu, Jeng-Feng Chiou, Hsun-Jin Jan, Hon-Jian Wei, Chung-Huei Hsu, Che-Tong Lin, Wen-Ta Chiu, Cheng-Wen Wu, Horng-Mo Lee, Win-Ping Deng

**Affiliations:** 1 Cancer Center and Section of Haematology-Oncology, Department of Internal Medicine, Taipei Medical University and Hospital, Taipei, Taiwan; 2 Cancer Center and Department of Radiation Oncology, Taipei Medical University and Hospital, Taipei, Taiwan; 3 Graduate Institute of Cell and Molecular Biology, Taipei, Taiwan; 4 Graduate Institute of Medical Sciences, Taipei Medical University, Taipei, Taiwan; 5 Department of Nuclear Medicine, Taipei Medical University, Taipei, Taiwan; 6 Graduate Institute of Oral Rehabilitation Sciences, Taipei Medical University, Taipei, Taiwan; 7 Wan-Fang Hospital and Taipei Medical University, Taipei, Taiwan; 8 Institute of Biomedical Sciences, Academia Sinica, Taipei, Taiwan; 9 Institute of Biomedical Materials and Engineering and Center of Excellence for Cancer Research (CECR), Taipei Medical University, Taipei, Taiwan

## Abstract

**Background:**

Invasiveness and metastasis are the most common characteristics of non small cell lung cancer (NSCLC) and causes of tumour-related morbidity and mortality. Mitogen-activated protein kinases (MAPKs) signalling pathways have been shown to play critical roles in tumorigenesis. However, the precise pathological role(s) of mitogen-activated protein kinase phosphatase-1 (MKP-1) in different cancers has been controversial such that the up-regulation of MKP-1 in different cancers does not always correlate to a better prognosis. In this study, we showed that the induction of MKP-1 lead to a significant retardation of proliferation and metastasis in NSCLC cells. We also established that rosiglitazone (a PPARγ agonist) elevated MKP-1 expression level in NSCLC cells and inhibited tumour metastasis.

**Methods:**

Both wildtype and dominant negative forms of MKP-1 were constitutively expressed in NSCLC cell line H441GL. The migration and invasion abilities of these cells were examined in vitro. MKP-1 modulating agents such as rosiglitazone and triptolide were used to demonstrate MKP-1's role in tumorigenesis. Bioluminescent imaging was utilized to study tumorigenesis of MKP-1 over-expressing H441GL cells and anti-metastatic effect of rosiglitazone.

**Results:**

Over-expression of MKP-1 reduced NSCLC cell proliferation rate as well as cell invasive and migratory abilities, evident by the reduced expression levels of MMP-2 and CXCR4. Mice inoculated with MKP-1 over-expressing H441 cells did not develop NSCLC while their control wildtype H441 inoculated littermates developed NSCLC and bone metastasis. Pharmacologically, rosiglitazone, a peroxisome proliferator activated receptor-γ (PPARγ) agonist appeared to induce MKP-1 expression while reduce MMP-2 and CXCR4 expression. H441GL-inoculated mice receiving daily oral rosiglitazone treatment demonstrated a significant inhibition of bone metastasis when compared to mice receiving sham treatment. We found that rosiglitazone treatment impeded the ability of cell migration and invasion *in vitro*. Cells pre-treated with triptolide (a MKP-1 inhibitor), reversed rosiglitazone-mediated cell invasion and migration.

**Conclusion:**

The induction of MKP-1 could significantly suppress the proliferative and metastatic abilities of NSCLC both in vitro and in vivo. Therefore, MKP-1 could be considered as a potential therapeutic target in NSCLC therapy and PPARγ agonists could be explored for combined chemotherapy.

## Background

Lung cancer represents the leading cause of cancer-related deaths in many countries. It is estimated that there are 0.2 million diagnosed cases of lung cancer and over 0.16 million patients die from it in the United States alone [1]. The most common type of lung cancer is non-small cell lung cancer (NSCLC) which contributes to over 75% of all cases [2]. Despite advances in therapeutic strategies, less than 15% of patients with NSCLC survive beyond 5 years of initial diagnosis. The precise etiology of NSCLC is complex. However, activating mutations of the *KRAS *protooncogene and/or *EGFR *are believed to play a major role in NSCLC tumorigenesis [3,4]. *KRAS *and *EGFR *mutations lead to overt activities of their downstream pro-survival and anti-apoptotic signaling pathways, attributing not only to the development of lung cancer but also a positive correlation to poor prognosis and response to conventional therapies [5]. Due to the extensive contributions from lateral signaling molecules and pathways, the therapeutic efficacies of KRAS and EGFR targeted therapies have been disappointing thus new regimens are required.

The mitogen-activated protein kinases (MAPKs) represent the common downstream effectors of EGFR and KRAS; the dynamic balance between MAPKs and their respective phosphatases (MKPs) ultimately determine whether cells undergo survival or apoptosis [6]. In particular, MKP-1, the prototype member of dual-specificity MAPK phosphatases, has been shown to play either pro-or anti-apoptotic role depending on the cell types. For instance, the over-expression of MKP-1 has been detected in breast cancer and associated to its malignancy [7]. On the contrary, decreased level of MKP-1 was found in association with high histological grade of prostate, colon and bladder cancer and distant metastasis [8]. In advanced epithelial ovarian cancer, this phosphatase was found down-regulated and its re-expression reduced the malignant potential of neoplasms [9]. Thus far, the role of MKP-1 in lung cancer has not been clearly illustrated. However, a previous study demonstrated an increased level of MKP-1 was accompanied with MAPKs in NSCLC lung specimens as compared to normal lung [10]. In the same report, the authors suggested that high MKP-1 expression levels independently predicted improved patients' survival. For these reasons, therapeutic strategies that employ MKP-1 induction for the suppression of tumor proliferation and metastasis could be of clinical importance. Several studies have demonstrated that PPARγ agonist, thiazolidinedione, a class of anti-diabetic drugs including rosiglitazone and troglitazone, significantly inhibit primary tumor growth and metastasis in a variety of cancers including colon, breast, prostate, brain and lung [11-15]. However, the mechanisms by which these PPARγ agonists suppress NSCLC cell tumorigenesis have not been elucidated fully. Based on these premises, we sought to explore the precise pharmacological role(s) of MKP-1 in the development and progression of NSCLC and the possible involvement of MKP-1 in rosiglitazone-mediated tumor suppression. To achieve our goals, we over-expressed either wildtype or dominant negative forms of MKP-1 in a NSCLC cell line, H441GL which contains dual reporter genes, enhanced green fluorescence protein (G) and firefly luciferase (L), to assess its impact both in vitro and in vivo, as well as the pharmacological induction of MKP-1 by rosiglitazone.

## Methods

### Reagents and Antibodies

Trizol regent, glutamine, gentamycin penicillin, and streptomycin were purchased from Life Technologies (Gaithersburg, MD) and all antibodies specific for MMP-2, MKP-1, phospho-p38, phospho-ERK, phospho-JNK, CXCR4 and α-tubulin were all purchased from Santa Cruz Biotechnologies (Santa Cruz, CA). Horseradish peroxidase-conjugated anti-rabbit IgG secondary antibody was purchased from Bio-Rad (Hercules, CA). Rosiglitazone powder was obtained from Dr. Wang CY from Far Eastern Memorial Hospital (supplied by GSK Taiwan branch) while rosiglitazone tablets, Avandia^® ^(4 mg) were purchased from Taipei Medical University Hospital Pharmacy. Dimethyl sulfoxide (DMSO) was used to dissolve both rosiglitazone powder and tables.

### Constructs, Cells and Culture Conditions

Non-small cell lung cancer (NSCLC) NCI-H441GL cells expressing dual reporters, enhanced green fluorescent protein (G) and firefly luciferase (L) were generously provided by Dr. Juri Gelovani at University of Texas, MD Anderson Cancer Center, Texas Houston, USA. Cells were grown in RPMI medium supplemented with 10% FBS, containing 2 mM glutamine and antibiotics (100 U/ml penicillin and 100 μg/ml streptomycin). After reaching 80% confluence, cells were treated with various concentrations of rosiglitazone and various inhibitors of MAPKs (30 μM, unless indicated) and incubated for 24 h (unless specified) in a 5% CO_2 _humidified incubator at 37°C. During rosiglitazone (0-30 μM) treatment, serum free medium was used in order to synchronize the cells and eliminate possible interference from serum. Both wildtype and dominant negative MKP-1 full length cDNAs were PCR-amplified with primers containing *BamH*I and *EcoR*I linkers and was inserted into pcDNA3.1 vector (cat #V79020, Invitrogen, Taipei, Taiwan). H441GL cells (CL1-5F4 and A549 cells) were seeded at 5 × 10^5 ^cells in 6-cm plates and were transduced with wildtype pcDNA3.1/MKP-1, dominant mutant pcDNA3.1/MKP-1CS (C258S mutation) and empty pcDNA3.1 vectors using Lipofectamine 2000 (Invitrogen, Taipei, Taiwan). After recovery, positive clones were selected using Geneticin (Gibco BRL Life Technologies, Inc.).

### RNA Extraction and Semi-Quantitative PCR Analysis

Total RNA was extracted from cultured cells using TRIzol according to manufacturer's protocols. Reverse transcription reactions were carried out to obtain cDNA according to standard protocols. Primer sequences are listed as follows (forward primer is listed first followed by the reverse primer). MKP-1 5'-GCTGTGCAGCAAACAGTCGA-3' and 5'-CGATTAGTCCTCATAAGGTA-3'; MMP-2 5'-GAGTTGGCAGTGCAATACCT-3' and 5'-GCCGTCCTTCTCAAAGTTGT-3'; Vimentin 5'-CTACATCGACAAGGTGCGCTT-3' and 5'-TGCCAGAGACGCATTGTCAA-3'; E-cadherin 5'-GACCATTCAGTACAACGACCCAACC-3' and 5'-ACCGCTTCCTTCATAGTCAAACACG-3'; CXCR4 5'-TTCTACCCCAATGACTTGTG-3' and 5'-ATGTAGTAAGGCAGCCAACA-3'; GAPDH 5'-GCCAAAAGGGTCATCATCTCTG-3' and 5'-CATGCCAGTGAG CTTCCCGT-3'; All primers were derived from human cDNA sequences, and PCR conditions were optimized so that the gene products were in the exponential phase of amplification (94°C for 2 min followed by 30 (30 sec) cycles at 94°C, 55°C for 1 min, and 72°C for 1 min; followed by a 7-min final elongation at 72°C). PCR products were resolved on 1.5% 2% agarose gels containing 1 μg/ml ethidium bromide and visualized/analyzed using FloGel-I (Fluorescent Gel Image System; TOP BIO Co.)

### Polyacrylamide gel electrophoresis and Western blotting

Cell lysates were separated by SDS-PAGE and transferred onto PVDF membranes for immunoblotting. Membranes were subject to blocking solution (1×PBS, containing 3% BSA and 0.1% Tween 20) for 1 h at room temperature followed by the incubation of respective primary and secondary antibodies. Immunodetection was carried out by LumiGLO chemiluminescence kit (Amersham, UK).

### Gelatin zymography

Equal numbers of cells (1 × 10^6^) were seeded onto 100-mm dishes and cultured with serum free medium for 24 h. Equal amount of media were collected and MMP-2 activities were determined by gelatin zymography with 0.1% gelatin as a substrate in a 10% SDS-polyacrylamide gel. After the addition of 2× sample buffer, cell media were directly loaded on to gels. Samples were renatured by exchanging SDS with 2.5% Triton X-100. The gel was incubated at 37°C in developing buffer containing 50 mM Tris-HCl, (pH 7.6) and10 mM CaCl2. Gel was then stained with 0.25% Coomassie blue R250, 40% methanol, and 10% acetic acid at room temperature and destained with 40% methanol, 10% acetic acid until the bands of lysis became clear. The MMP-2-relative photographic density was quantitated by scanning the photographic negatives on a gel analysis system (BioSpectrumAC Imaging System Vision with LS software, UVP Inc., Upland, California, USA).

### In Vitro Invasion Assay

Matrigel invasion assays were performed with a Boyden-chamber assay using BD BioCoat Matrigel Invasion Chambers (BD Biosciences, San Jose, CA). To determine the invasiveness, H441GL, H441GL/MKP-1 and H441GL/MKP-1CS cells were seeded in the upper compartment of each chamber (1 × 10^5^cells/well, serum-free DMEM) while the lower compartments were filled with DMEM containing 10% FCS. Non-invading cells were gently removed after 24 h. Cells on the bottom side of the filter were fixed, stained, and counted under a light microscope (×200 magnification). All experiments were repeated three times.

### In Vitro Cell Viability Assay

For dye exclusion assay, cells were seeded at the density of 2.5 × 10^4 ^cells/well, in 24-well plates. The media were removed, and the cells were rinsed with PBS before incubation with trypsin. Cells were then washed, and resuspended in 0.4% trypan blue, and live cells were counted using a hemocytometer.

### In vivo monitoring of NSCLC

SCID (Severe combined immunodeficiency) mice were maintained in a specific pathogen-free environment in compliance with institutional policy and all animal procedures were previously approved by the IACUC (Institutional Animal Care and Use Committee) at Taipei Medical University. Wildtype NSCLC H441GL and MKP-1 overexpressing H441GL (H441GL/MKP-1) were intravenously administered into SCID mice via tail vein at a concentration of 5.5 × 10^5^/100 μl PBS. The growth and metastasis of tumor cells were monitored in real time with IVIS 200 imaging system, equipped with Living Imaging software (Xenogen, Alameda, CA). Rosiglitazone tablets (Avandia™, 4 mg) was grounded and dissolved in 0.1% DMSO and given to mice bearing H441GL tumor via daily gavage at a concentration of 10 mg/kg/d.

### Statistical Analysis

All data are expressed as the means ± SD for the number of experiments. Statistical significance (*, *p *< 0.05; **, *p *< 0.01) between experimental and control groups was calculated by student T-test.

## Results

### Induction of MKP-1 Reduces Cell Proliferation Rate and Initiates Mesenchymal-to-Epithelial Switch in NSCLC Cells

**Figure 1:**
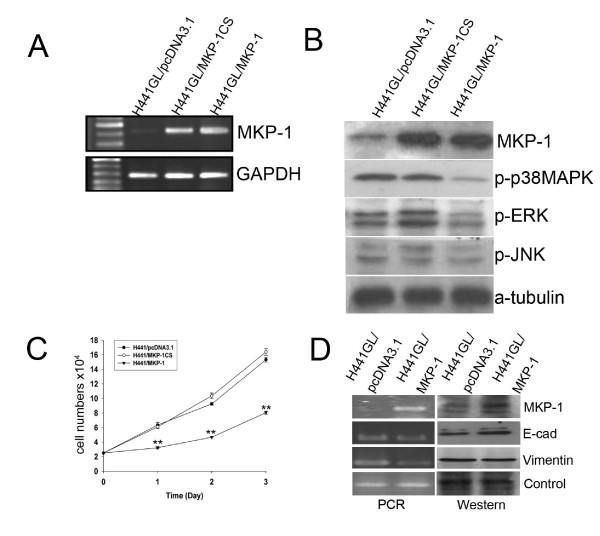
**In Vitro Characterizations of MKP-1 over-expressing H441GL NSCLC Cells (A) In vitro validation of MKP-1 over-expression in H441GL cells at transcriptional level**. Both dominant negative (H441GL/MKP-1CS) and wildtype MKP-1 (H441GL/MKP-1) constructs were transduced into H441GL cells Strong MKP-1 transcripts were visualized by ethidium bromide stain Empty vector pcDNA31 served as a control and demonstrated no increased amount of MKP-1 transcript in the tranduced host GAPDH served as a loading control (B) Immunoblots showing MKP-1 over-expression reduced MAPK activities H441GL cell lysates were immunoblotted with antibodies specific for MKP-1, phosphorylated p38MAPK, ERK, JNK or α-tubulin Phosphorylated MAPKs including p38MAKP, ERK and JNK activities were down-regulated by wildtype MKP-1 over-expression (C) Cellular proliferation was examined in MKP-1 over-expressing H441GL cells using dye exclusion assay H441GL cells transduced with dominant negative MKP-1 (H441/MKP-1CS) and empty vector (H441GL/pcDNA31) exhibited similar proliferation rate whereas wildtype MKP-1 over-expressing H441GL cells (H441GL/MKP-1) demonstrated markedly reduced proliferation rate Data was shown as mean ± SD of three independent experiments (D) Induction of MKP-1 triggered mesenchymal-to-epithelial status switch in H441GL cells by increasing E-cadherin (E-cad) while decreasing Vimentin expression Internal controls used in PCR and Western analyses were GAPDH and α-tubulin respectively.

To examine the role of MKP-1 in the development and progression of NSCLC, H441GL cells were selected for transient over-expression of both wildtype (H441GL/MKP-1) and non-functional MKP-1 (H441GL/MKP-1CS). Both transcriptional and translational levels of *mkp-1 *in H441GL cells were significantly up-regulated (Figure 1A and 1B), indicating a successful expression of *mkp-1 *gene. Next, we examined all three major players involved in MAPK signal transduction pathway, namely p38, ERK1/2 and JNK. An increased level of wildtype MKP-1 resulted in a decreased level of phospohorylated p38 and ERK1/2 and JNK to a much less extent. Conversely, over-expression of non-functional MKP-1 had no effect on the 3 members of MAKP pathway (Figure 1B, all see Additional file 1). Because MAPKs are major cell cycle regulators, cellular proliferation rates of H441GL/MKP-1 (wildtype) and H441GL/MKP-1CS (dominant negative mutant) were examined and compared. H441GL/MKP-1 cells exhibited a marked reduction in proliferation rate when compared to its mutant and mock-transfected counterparts (Figure 1C). Similarly, MKP-1 over-expression also led to a reduction in cell viability in other NSCLC cell lines namely CL1-5 F4 and A549 cells (see Additional file 2).

An increase in MKP-1 protein expression also initiated a mesenchymal-to-epithelial switch in H441GL cells. A decrease in vimentin transcript was accompanied by a slight increase in E-cadherin transcript, in MKP-1 over-expressing H441GL cells (Figure 1D).

### Induction of MKP-1 Down-regulates MMP-2 and CXCR4 Expression in NSCLC H441GL Cells

**Figure 2:**
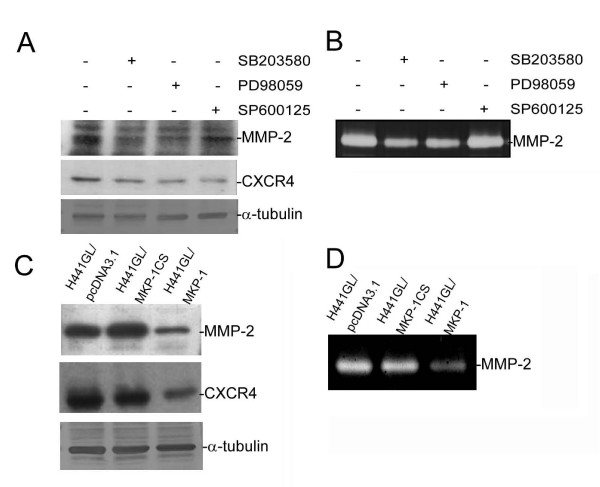
**MKP-1 regulates expressions of MMP-2 and CXCR4 in H441GL cell**. H441GL cells were treated with a p38MAPK (SB203580), an ERK1/2 inhibitor (PD98059), or a JNK (SP600125) (all at 30 μM) (A) Cell lysates were immunoblotted with antibodies specific for MMP-2, CXCR4, or α-tubulin Inhibitors against MAKPs all reduced both CXCR4 and MMP-2 expression levels except inhibitor against JNK did not significantly reduced MMP-2 expression Corresponding MMP-2 activity was measured using zymography (B). (C) MMP-2 and CXCR4 expression were examined in MKP-1 over-expressing H441GL cells Decreased expression levels of both molecules were detected in wildtype MKP-1 over-expressing H441GL cells (H441GL/MKP-1) when compared to those of in vector control (H441GL/pcDNA31) and dominant negative form of MKP-1 (H441GL/MKP-1CS) Corresponding MMP-2 enzymatic activity was demonstrated by zymography (D).

The ability of tumour cells to invade and migrate has been ascribed to the up-regulation of matrix metalloproteases (MMPs) and chemotactic axis CXCR4/SDF-1 [16]. In addition, MAPKs activities have been linked to the regulation of MMPs and CXCR4 [17,18]. Initially, we established that inhibition of MAPKs led to the down-regulation of MMP-2 and CXCR4. As depicted in Figure 2A, H441GL cells treated with SB203580 (a p38 MAPK inhibitor), PD98059 (an ERK inhibitor), or SP600125 (a JNK inhibitor), showed reduced expression levels of MMP-2 and CXCR4 when compared to control cells. Similarly, the inhibition of p38 MAPK, ERK and JNK led to a reduced MMP-2 enzymatic activity in H441GL cells (Figure 2B). It appeared that MMP-2 enzymatic activity was hampered to a larger extent when p38 MAPK and ERK were inhibited. Next, we demonstrated that MKP-1 over-expression directly resulted in a suppressed enzymatic activity of MMP-2 (Figure 2D) and was accompanied with a lower expression level of MMP-2 and CXCR4 (Figure 2C) in H441GL cells. The catalytically inactive mutant of MKP-1 (H441GL/MKP-1CS) did not affect MMP-2 and CXCR4 expressions.

### Increased MKP-1 Expression Reduces Invasive and Migratory Abilities of H441GL Cells

**Figure 3:**
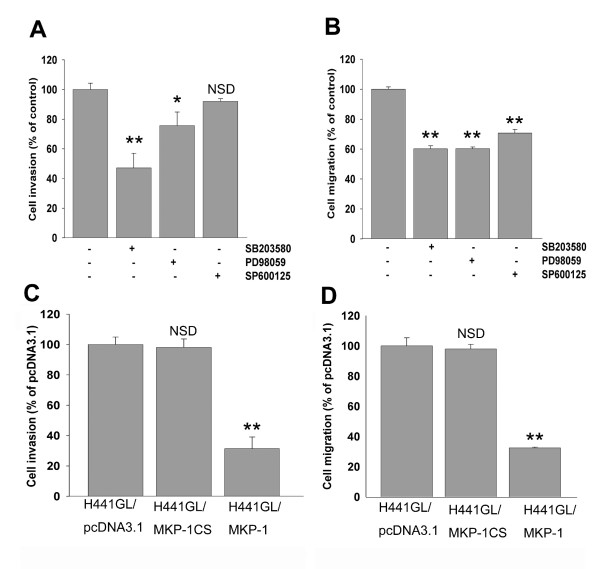
**MKP-1 regulates invasive and migratory abilities in NSCLC H441GL cells**. Cell invasive (A) and migratory (B) abilities of H441GL cells were examined under the presence of different inhibitors of MAPKs (all at 30 μM) including p38 MAPK (SB203580), ERK1/2 (PD98059), and JNK (SP600125) inhibitors The degree of inhibition in H441GL invasiveness (C) and migration (D) when MKP-1 was over-expressed was examined The inhibition of invasiveness and migration was found at the following order, p38>ERK>JNK and p38≃ERK>JNK respectively Data represent mean ± SD (n = 3).

We established that MAPK pathways were responsible for regulating the expression levels of MMP-2 and CXCR4, concomitantly MKP-1 expression was negatively correlated to the expression and activity of both gene products. It was then our goal to demonstrate that MAPKs and MKP-1 regulated cellular invasiveness and migration via MMP-2 and CXCR4. H441GL cells treated with MAPK pathway inhibitors, SB203580, PD98059, and SP600125, exhibited a reduced level of invasiveness (Figure 3A) and migration (Figure 3B) in H441GL cells. It appeared that both cellular invasiveness and migration were hampered to a greater extent in H441GL cells when p38 MAPK and ERK were both inhibited (Figure 3A and 3B). Subsequently, H441GL cells were transduced with MKP-1 and examined its roles in cellular invasiveness and migration. It was observed that both invasive and migratory (Figures 3C and 3D, respectively) abilities of MKP-1 over-expressing H441GL cells were severely affected. These observations were in agreement with the reduced expression levels of MMP-2 and CXCR4 as the result of an increase in MKP-1 expression. Similarly, when MKP-1 expression level was elevated in A549 and CL1-5F4 (a highly invasive lung adenocarcinoma cell line) cells, their invasiveness was also significantly reduced (see Additional file 3).

### Pharmacologically-induced MKP-1 Expression Leads to the inhibition of Invasion and Migration of H441GL cells In Vitro

**Figure 4:**
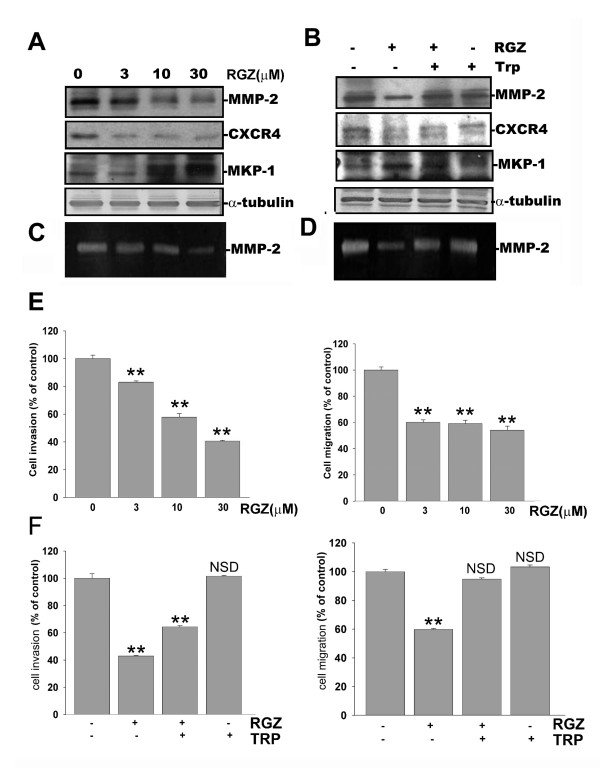
**Rosiglitazone regulates MMP-2 and CXCR4 expression via MKP-1 induction**. (A) H441GL cells treated with different concentrations of rosiglitazone (RGZ, 0 to 30 μM) demonstrated a decrease in both MMP2- and CXCR4 protein expression levels accompanied by an increase in MKP-1 expression level in a dose-dependent fashion This effect was reversed when triptolide (TRP, a MKP-1 inhibitor at 10 ng/ml) was added (B) (C-D) As demonstrated by zymography, MMP-2 enzymatic activity was reduced by RGZ in a dose-dependent manner, and this reduction by RGZ could be reversed by the addition of triptolide (E-F) Corresponding in vitro invasion and migration assays also showed RGZ treatment decreased cellular mobility of H441GL cells Data expressed as mean ± SD (n = 3).

Rosiglitazone (RGZ), a PPARγ agonist used in type 2 diabetes treatment, has been shown to reduce the malignancy in variety of cancers [12]. In rosiglitazone-treated HUVECs cells, the expression and activity of ERK1/2 and p38 MAPK were significantly down-regulated [19]. To this end, we set out to determine whether rosiglitazone is a pharmacological inducer of MKP-1 in NSCLC model. In NSCLC, rosiglitazone treatment was found to increase the expression of MKP-1 in H441GL cells in a dose-dependent manner (Figure 4A). This dose-dependent rosiglitazone-mediated up-regulation of MKP-1 expression was accompanied by a reduction in MMP-2 and CXCR4 expression level (Figure 4A) and activity (Figure 4C). When MKP-1 was inhibited by a pharmacological inhibitor, triptolide (Trp), rosiglitazone-mediated down-regulation of MMP-2 and CXCR4 was hampered as the result of diminished level of MKP-1 (Figure 4B and 4D). Next, a matrigel-coated chamber system was used to investigate the effects of rosiglitazone on H441GL invasiveness and migration. Rosiglitazone posed an inhibitory effect on both invasive (Figure 4E left panel) and migratory (Figure 4E right panel) abilities of H441GL cells (see Additional file 4). The highest concentration of rosiglitazone (30 μM) reduced H441GL cells' invasive and migratory abilities by approximately 60% and 40%, respectively. When triptolide was added rosiglitazone-mediated reduction in invasion and migration was reversed (Figure 4F, left and right panels, respectively).

### Elevated MKP-1 Expression, Intrinsic or Pharmacologically-induced, Inhibits NSCLC Metastasis in Mice

**Figure 5:**
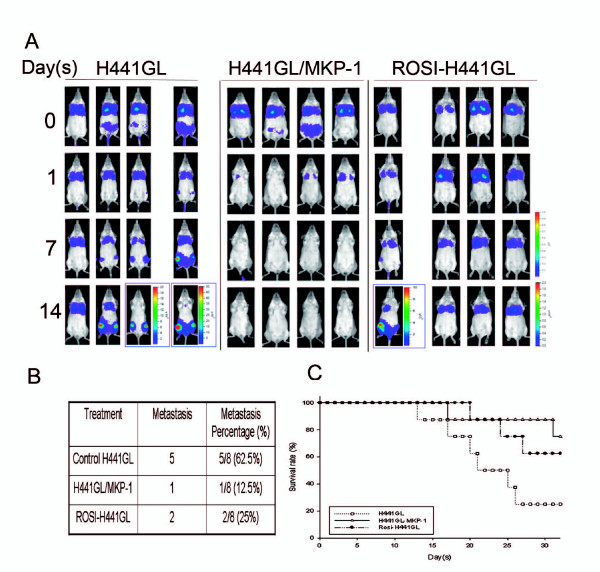
**Non-invasive bioluminesence monitoring of NSCLC tumour suppression by either MKP-1 over-expression or rosiglitazone treatement**. (A) Representative bioluminescence images of SCID mice injected with either control H441GL cells (H441GL), MKP-1 over-expressing H441GL (H441GL/MKP-1) or control H441GL-inoculated mice receiving daily oral rosiglitazone (100 mg/kg/d) treatment (ROSI-H441GL) The intensity of bioluminescent signals increased in both control and ROSI-treated groups but not the mice inoculated with H441GL/MKP-1, over the 14-day observation period The intensity of bioluminescent signals reflects the increase in tumour burden (B) Metastatic ability was tabulated based on bioluminescence analysis Control H441GL mice showed the highest lung-to-hind leg metastasis rate (625%) followed by ROSI-H441GL (25%) and H441GL/MKP-1 animals (125%) (C) Kaplan-Meier plot indicated that H441GL/MKP-1 animals had the best survival rate among the three groups, ROSI-H441GL group being the modest (60%) and the control H441GL animals the worst (25%) The insert table depicts the percentage of lung-to-bone metastases obtained from three groups.

The physiological roles of MKP-1 in NSCLC tumorigenesis and metastasis were further explored in vivo, assisted by non-invasive molecular imaging system. Mice, inoculated with wildtype H441GL cells, developed large tumour burden and signs of distant metastasis within a week. By week 2, most mice showed severe lung-bone metastasis (Figure 5A, left panels). In contrast, animals received MKP-1 over-expressing H441GL cells did not even develop tumour burden and remained tumour-free after two weeks (Figure 5A, middle panels, also see Additional file 5). In parallel, we examined whether rosiglitazone's in vitro anti-proliferative and anti-metastatic effects could be replicated in H441GL-inoculated mice by oral gavage treatment. Mice receiving daily oral of gavage rosiglitazone, developed tumour burden one week post-inoculation (Figure 5A, right panels). However, lung-bone metastasis appeared to be significantly delayed in the rosiglitazone-treated animals. The first incidence of metastasis was not observed until week 2 and most animals remained metastasis-free. The rate of metastasis was tabulated based on our optical imaging analysis. As indicated in Figure 5B, MKP-1 over-expressing group exhibited the lowest metastasis rate (12.5%), followed by rosiglitazone-treated group (25%) and control group (62.5%). The survival rate of these animals was recorded and represented by a Kaplan-Meier plot after one month of inoculation (Figure 5C). Mice inoculated with H441GL/MKP-1 cells exhibited the highest survival rate (80%) followed by the animals receiving rosiglitazone treatment (60%) while the control animals without any treatment had the lowest survival rate (25%).

## Discussion

Herein, we assessed the functional significance of MKP-1 in the regulation of tumor proliferation and metastasis using a mouse model assisted by molecular imaging technology. An increased level of MKP-1 by protein over-expression significantly slowed the progression of lesions from premalignant to invasive and impaired the growth of tumours. Moreover, we provided mechanistic evidence that MKP-1 suppresses both tumour proliferation and metastasis via promoting mesenchymal-to-epithelial transition followed by the dampening of MMP-2 and CXCR4 activities. As for clinical translation, rosiglitazone, a widely used anti-diabetic agent was shown to convey its anti-tumour effects via the induction of MKP-1.

Mitogen-activated protein kinase dual specificity phosphatase-1 (primarily known as MKP-1, DUSP1 or CL100) is an immediate-early gene whose expression is under the regulation of various cellular signals [20-22]. MKP-1 negatively regulates its substrates, mitogen-activated protein kinases (MAPKs) including p38MAPK, JNK and ERK1/2, via dephosphorylation [23]. MKP-1 expression level has been positively linked to different types of cancer such as human ovarian carcinoma, breast and prostate cancer [7,24,25]. However, a negative correlation between MKP-1 expression and the prognosis of cancer has also been documented in hepatocellular carcinomas [26,27]. In the case of lung cancer, the role of MKP-1 appears to be controversial as well. It was shown that a higher MKP-1 expression was detected in NSCLC versus small cell lung cancer cell lines. However, there is no definitive correlation between individual clinicopahtological variables or MAPK phosphorylation status when examined by immunohistochemical assay and MKP-1 expression [11]. In order to better define the role of MKP-1 in NSCLC tumorigenesis, we over-expressed MKP-1 in H441GL cells. It was found that the increased MKP-1 expression level significantly retarded both proliferative and metastatic abilities in these cells both in vitro and in vivo and these inhibitory effects were attributed to the combined and differential dephosphorylation of three major MKP-1's substrates in the decreasing order of preference, p38MAPK, ERK1/2 and JNK. The selective hyperactivation of p38 MAPK signalling in NSCLC (and other cancer types) has been correlated to malignant transformation [28,29] via the induction of MMP-2 [30-32] and CXCR4/SDF-1 chemotaxis axis [33]. In addition, the deactivation of ERK 1/2 and JNK is associated with cell cycle arrest [34] and impaired cell migration [35,36] respectively. Thus, the decreased malignancy of H441GL/MKP-1 appeared to be logical and supported.

Interestingly, the phosphorylation level of JNK (the activated form) was not significantly altered in H441GL/MKP-1 cells. Currently, the underlying mechanism of this selective MAPK signalling down-regulation in H441GL/MKP-1 cells remains elusive but it could be attributed to the preferential up-regulation of p38 MAPK in NSCLC [29] and the comparatively lesser role of JNK in tumorigenesis to p38 MAPK [37]. As an added advantage, MKP-1 over-expressing NSCLC cells displayed a significant reduction in glucose uptake ability, a reduced Warburg effect (see Additional file 6). This significantly reduced glucose uptake ability of H441GL/MKP-1 cells provides a further support to the observed proliferation suppression both in in vitro and bioluminescence imaging data where mice inoculated H441GL/MKP-1 cells did not result in tumorigenesis. Similarly, rosiglitazone treatment also negatively affected H441GL cells' glucose uptake ability although to a lesser extent when compared to intrinsic elevation of MKP-1 in H441GL/MKP-1 cells. This could partially explain why oral rosiglitazone treatment was not as effective as MKP-1 over-expression in tumour suppression.

**Figure 6:**
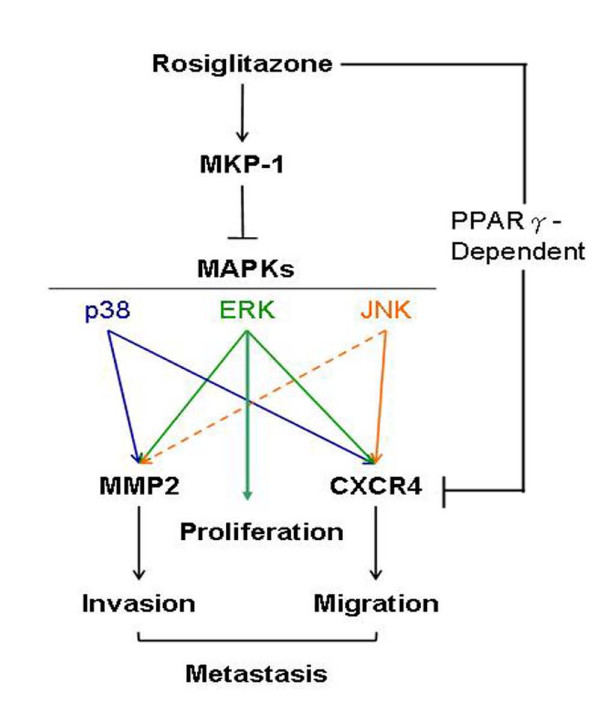
**Proposed pharmacological role of MKP-1 in rosiglitazone-mediated tumour suppression**. Rosiglitazone exerts its anti-tumour effects via both PPARγ-dependent (via CXCR4) and PPARγ-independent (via MKP-1 induction) pathways Two major MAPKs affected by the induction of MKP-1 are p38 and ERK which participate in the regulation of cellular mobility and proliferation respectively (→ direct stimulatory modification; --> tentative stimulatory modification; --| direct inhibitory modification).

Peroxisome proliferator-activated receptor-gamma (PPARγ) exerts compounded roles in cell differentiation, tissue metabolism and host immunity and recently is implicated in tumor suppression [38]. Of clinical significance, a 33% reduction in lung cancer risk in diabetic patients who received thiazolidinedione class drugs was observed [39]. However, the role of PPARγ in tumorigenesis have been controversial [40-43] and the molecular mechanism underlying PPARγ-mediated tumor suppression remains unclear [44,45]. In this study, we demonstrated that the usage of rosiglitazone (a PPARγ agonist) inhibited NSCLC H441GL cell growth and metastasis both in vitro and in vivo. Based on our experimental data, we proposed that rosiglitazone-induced tumor suppression is due to a combination of PPARγ-dependent and PPARγ-independent pathways. Suppression of tumor growth is most likely achieved by the induction of MKP-1 which leads to the down-regulation of p38MAPK and ERK1/2, a PPARγ-independent event; retardation of metastases is via a PPARγ-dependent pathway which directly decreases CXCR4 and MMP expressions (Figure 6). We also found that rosiglitazone treatment resulted in altered expression levels in other genes using a RNA array system. The expression levels of bone morphogenetic proteins 2 and 4 (BMP2 and BMP4), both have been suggested to play important roles in tumour metastasis, showed a 10 and 14 fold decrease by rosiglitazone, respectively. In contrast, rosiglitazone treatment elicited a 14 fold increase in tumour suppressor gene INK4a expression (Data not shown).

Important insights were obtained from our H441GL-inoculated mice using non-invasive bioluminescence imaging. First, MKP-1 over-expressing H441GL-inoculated animals (H441GL-MKP-1) exhibited a much higher survival rate when compared to both rosiglitazone-treated and sham-treated animals. In fact, bioluminescence imaging data revealed that tumour burden was reduced significantly in H441GL-MKP-1-inoculated mice one week post inoculation, indicating that an increase in MKP-1 expression in H441GL cells prevented tumour growth in vivo. In addition, this observation established that an intrinsic up-regulation of MKP-1 represented a powerful tool for tumour suppression and a potential candidate for target therapy development. Second, H441GL-bearing mice receiving rosiglitazone (100 mg/kg/d) treatment (Rosi-H441GL) exhibited similar tumour volume when compared to control H441GL group but distant (bone) metastasis in Rosi-H441GL group was significantly inhibited. Our data was supported by an earlier study demonstrating oral rosiglitazone treatment also inhibited metastasis of murine mammary cancer cells without affecting the tumour size [46]. However, this finding revealed that the cellular level of MKP-1 induced by current rosiglitazone treatment might not be sufficient, perhaps due to the difference between the bioavailability, to mirror our in vitro observations. Similarly, a recent report indicated that lung cancer-bearing mice which received lone oral rosiglitazone treatment (chow pellet) did not produce as a significant degree of tumour suppression as compared to intraperitoneal injected carboplatin or combined rosiglitazone and carboplatin treatments [47].

## Conclusion

Treating lung cancer is a notoriously difficult task due to its high tendency for distant metastasis and resistance to both chemo- and radiotherapies. Despite the fact that MKP-1 has been implicated as a negative prognostic indicator in several cancers including ovarian [26] and breast [48] carcinomas, there are also incidences where over-expression of MKP-1 appears to be beneficial as in hepatocellular [27] and urothelial carcinomas [49]. In this study, we presented a collection of evidence supporting MKP-1's role as a tumour suppressor in NSCLC. An elevated level of MKP-1 protein either by over-expression or rosiglitazone treatment, resulted in the suppression of proliferative, migratory and invasive abilities of H441GL cells. Using molecular imaging technique, we were able to in vivo monitor H441GL tumour progression where MKP-1 over-expressing and rosiglitazone-treated groups demonstrated significant tumour growth and metastasis inhibition respectively as compared to the wildtype H441GL inoculated group. As for pharmacological relevance, we reported that rosiglitazone, a widely-used and well-tolerated anti-diabetic agent, conveys its anti-tumour ability via MKP-1 induction. Based on these premises, MKP-1 itself could be a candidate for target therapy and agents capable of inducing MKP-1 expression such as rosiglitazone or other similar compounds should receive considerations for clinical tests.

## Abbreviations

NSCLCL: non-small cell lung cancer; MKP-1: mitogen activated protein kinase phosphatase-1; RGZ: rosiglitazone; Trp: triptolide.

## Competing interests

The authors declare that they have no competing interests.

## Authors' contributions

CJT, ATHW, HML, HJJ, JFC and WPD were involved in experimental designs. ATHW, CJT and HJJ and were involved in manuscript writing. ATHW HJJ and HJW performed the experiments. HJW, CHH and CTL were involved in optical imaging analysis. WTC and CWW contributed to manuscript preparation. All authors read and approved the final manuscript.

## Pre-publication history

The pre-publication history for this paper can be accessed here:

http://www.biomedcentral.com/1471-2407/10/95/prepub

## Supplementary Material

Additional file 1 **Figure S1**. Major MAKP protein expression profiles in MKP-1 and MKP-1CS over-expressing H441GL cells The total protein levels of p38MAPK, ERK and JNK were not affected by the over-expression of MKP-1 as demonstrated by the western blots. Click here for file

Additional file 2 **Figure S2**. MKP-1 induction reduces NSCLC viability Two other NSCLC cell lines CL1-5F4 and A549 over-expressing MKP-1 were also examined for their viability using MTT assay Cells with elevated MKP-1 expression level, CL1-5F4/MKP-1 and A549/MKP-1 showed a significantly lower viability when compared to their respective vector-transduced controls, CL1-5F4/pcDNA31 and A549/pcDNA31 (n = 3). Click here for file

Additional file 3 **Figure S3**. MKP-1 over-expression reduces invasiveness in CL1-5F4 and A549 NSCLC cells Both CL1-5F4 and A549 were also examined for their in vitro invasive ability using matrigel assay (A) Photographic representations of the cells migrated through the matrigel system (B) Quantitative analysis of cell invasiveness Both CL1-5F4/MKP-1 and A549/MKP-1 cells appeared to lose their invasive ability under the influence of MKP-1 over-expression while the vector controls remain highly invasive (n = 3). Click here for file

Additional file 4 **Figure S4**. Cell viability assay of H441GL cells treated with various concentrations of rosiglitazone (RGZ) The MTT survival ratio of H441GL cells were obtained at different RGZ concentrations (ranging from 0-30 μM). RGZ appeared to have a minor role in affecting cellular viability because even at 30 μM, the survival ratio was still maintained approximately at 87%. The slight decrease in the cell viability in RGZ treated H441GL cells did not contribute to the marked effect of RGZ in reducing invasiveness and migration in these cells. Click here for file

Additional file 5 **Figure S5**. In vivo monitoring of tumorigenesis of H441GL/MKP-1CS (dominant negative) inoculated mice. Tumorigenesis in mice inoculated with H441GL cells expressing the dominant negative form of MKP-1 was observed using non-invasive bioluminescence imaging. As demonstrated, H441GL/MKP-1CS cells behaved very similarly to H441GL parental cells. Click here for file

Additional file 6 **Figure S6**. Glucose uptake ability is down-regulated by the induction of MKP-1. Glucose uptake ability was measured and represented by the percentage of radio-active ^3^H-glucose incorporated in different NSCLC cell lines (A) Glucose uptake ability was severely retarded in all MKP-1 over-expressing cells (MKP-1) when compared with their respective controls (pcDNA31) (B) Treatment of rosiglitazone (30 μM) also suppressed glucose uptake ability in ROSI/A549 and ROSI/H441GL cells but not their respective controls (All experiments were performed in triplicates). Experimental protocol for glucose uptake assay. Cancer cells have been known to exhibit enhanced metabolism, as reflected in the significantly marked glucose uptake ability (Warburg effect). Based on our finding in this study, MKP-1 over-expressing NSCLC cells showed a marked reduction in proliferative ability via ERK-mediated pathway. To further support this notion, we examined the glucose uptake ability of these MKP-1 over-expressing NSCLC cells. Glucose uptake assay was performed in triplicate. NSCLC cells were seeded in 12-well plate at a density of 3 × 10^5^cells/well. Cells were incubated in medium contained 0.1 mM 2-deoxy-D-glucose and 0.5 μCi 2- [1,2-^3^H]-2-deoxy-D-glucose. Following incubation for 1 h, samples were washed twice with cold Ca^2+^- and Mg^2+^-free phosphate-buffer saline. Cells were then lyzed in 10 mM Tris-HCl (pH 8.0) containing 0.2% SDS, the incorporated radioactivity was then determined using TopCount NXT™ Microplate Scintillation and Luminescence Counter (Perkin Elmer, Taipei, Taiwan). Click here for file
