# Supplementary figures and images for: The investigation of Mitogen-Activated Protein kinase Phosphatase-1 as a potential pharmacological target in non-small cell lung carcinomas, assisted by non-invasive molecular imaging
Source: BMC Cancer. 2010 Mar 12;10:95. doi: 10.1186/1471-2407-10-95 (PMC2850900; doi:10.1186/1471-2407-10-95)

H441GL/pcDNA3.1

H441GL/MKP-1 CS

H441GL/MKP-1

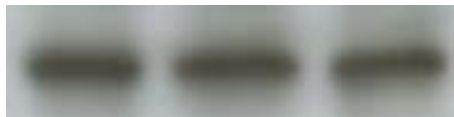

p38MAPK

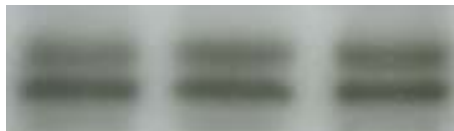

ERK

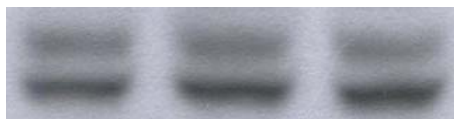

JNK

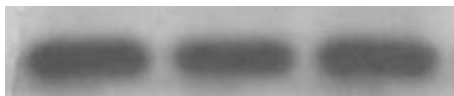

$\alpha$ -tubulin

Supplement: Additional file 1 — Figure S1. Major MAKP protein expression profiles in MKP-1 and MKP-1CS over-expressing H441GL cells The total protein levels of p38MAPK, ERK and JNK were not affected by the over-expression of MKP-1 as demonstrated by the western blots. [file 1471-2407-10-95-S1.PDF]

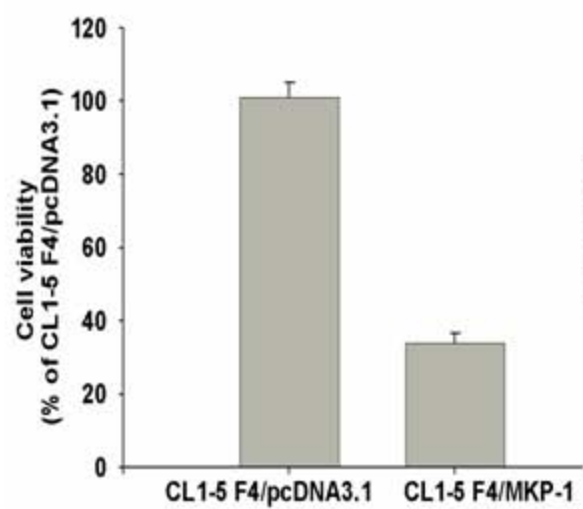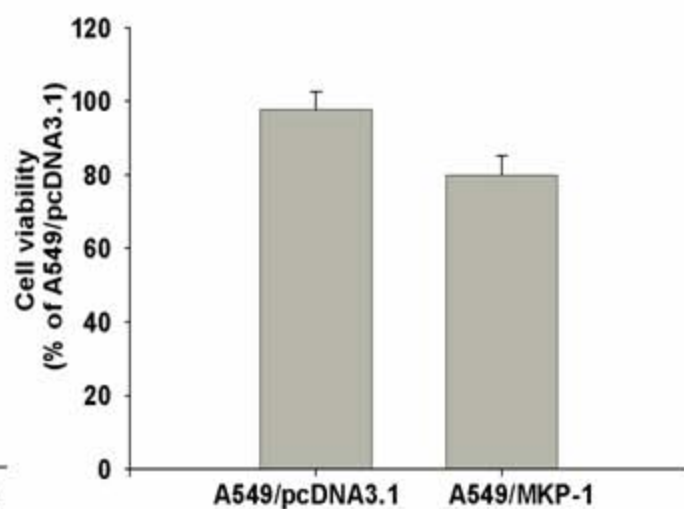

Supplement: Additional file 2 — Figure S2. MKP-1 induction reduces NSCLC viability Two other NSCLC cell lines CL1-5F4 and A549 over-expressing MKP-1 were also examined for their viability using MTT assay Cells with elevated MKP-1 expression level, CL1-5F4/MKP-1 and A549/MKP-1 showed a significantly lower viability when compared to their respective vector-transduced controls, CL1-5F4/pcDNA31 and A549/pcDNA31 (n = 3). [file 1471-2407-10-95-S2.PDF]

A

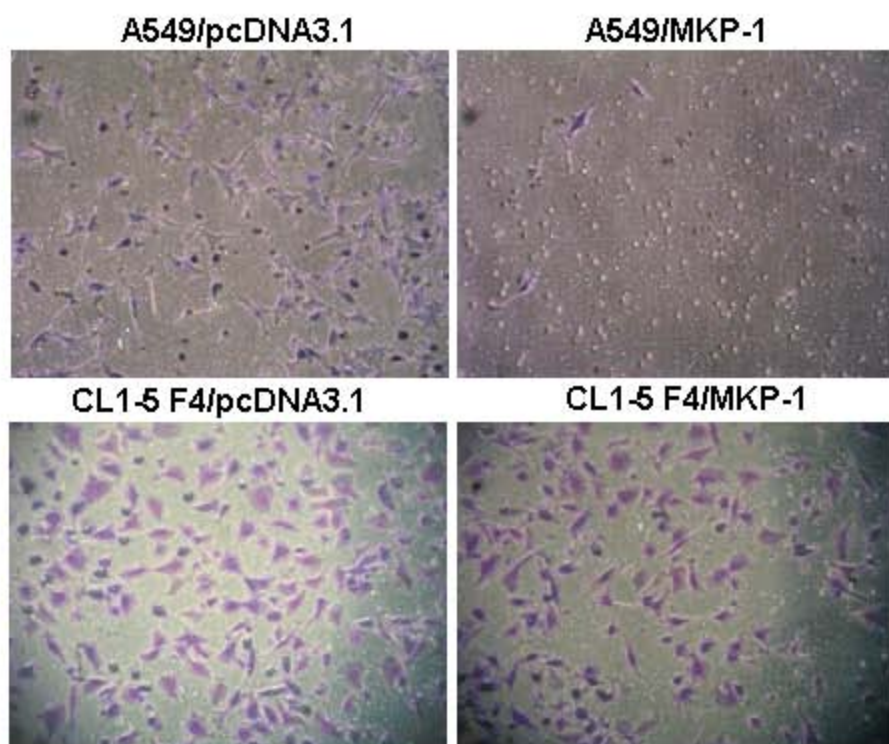

B

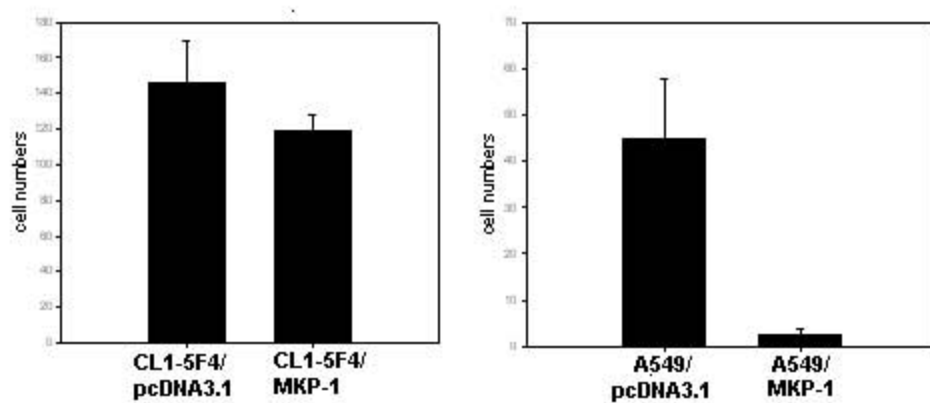

Supplement: Additional file 3 — Figure S3. MKP-1 over-expression reduces invasiveness in CL1-5F4 and A549 NSCLC cells Both CL1-5F4 and A549 were also examined for their in vitro invasive ability using matrigel assay (A) Photographic representations of the cells migrated through the matrigel system (B) Quantitative analysis of cell invasiveness Both CL1-5F4/MKP-1 and A549/MKP-1 cells appeared to lose their invasive ability under the influence of MKP-1 over-expression while the vector controls remain highly invasive (n = 3). [file 1471-2407-10-95-S3.PDF]

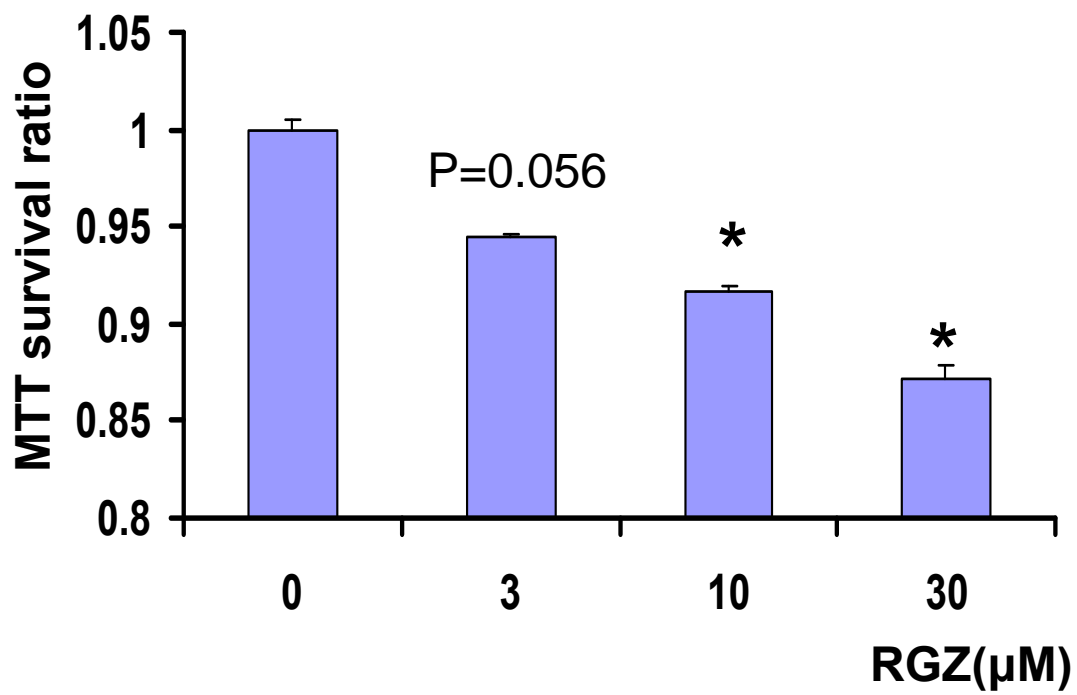

Supplement: Additional file 4 — Figure S4. Cell viability assay of H441GL cells treated with various concentrations of rosiglitazone (RGZ) The MTT survival ratio of H441GL cells were obtained at different RGZ concentrations (ranging from 0-30 μM). RGZ appeared to have a minor role in affecting cellular viability because even at 30 μM, the survival ratio was still maintained approximately at 87%. The slight decrease in the cell viability in RGZ treated H441GL cells did not contribute to the marked effect of RGZ in reducing invasiveness and migration in these cells. [file 1471-2407-10-95-S4.PDF]

Day 0

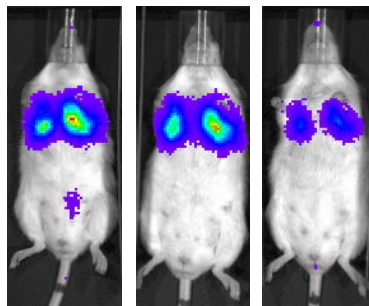

Day 1

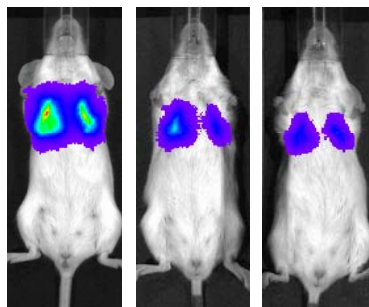

Day 7

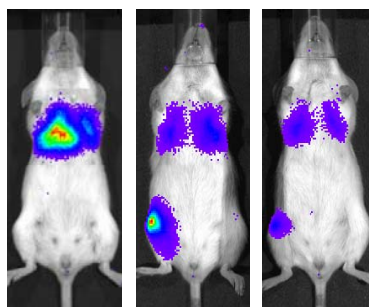

Day 14

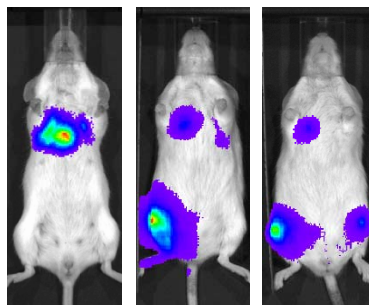

Day 0 ~ 7

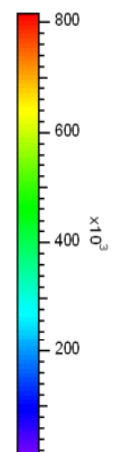

Day 14

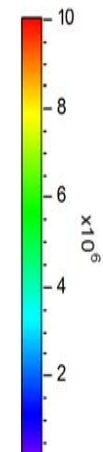

Supplement: Additional file 5 — Figure S5. In vivo monitoring of tumorigenesis of H441GL/MKP-1CS (dominant negative) inoculated mice. Tumorigenesis in mice inoculated with H441GL cells expressing the dominant negative form of MKP-1 was observed using non-invasive bioluminescence imaging. As demonstrated, H441GL/MKP-1CS cells behaved very similarly to H441GL parental cells. [file 1471-2407-10-95-S5.PDF]

**A**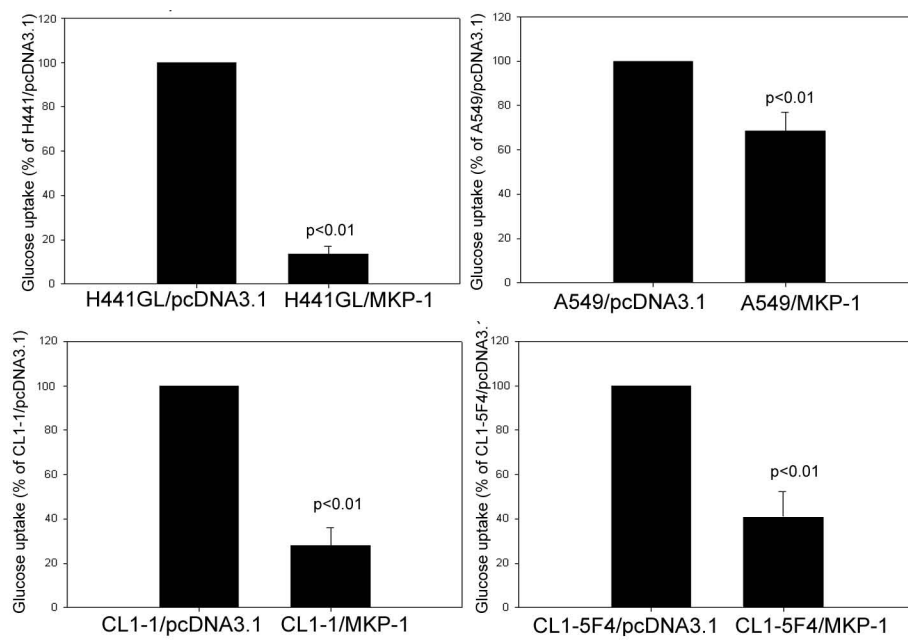**B**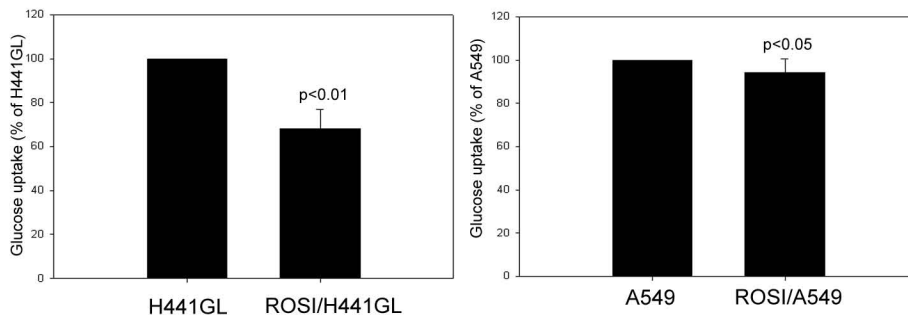

Supplement: Additional file 6 — Figure S6. Glucose uptake ability is down-regulated by the induction of MKP-1. Glucose uptake ability was measured and represented by the percentage of radio-active 3H-glucose incorporated in different NSCLC cell lines (A) Glucose uptake ability was severely retarded in all MKP-1 over-expressing cells (MKP-1) when compared with their respective controls (pcDNA31) (B) Treatment of rosiglitazone (30 μM) also suppressed glucose uptake ability in ROSI/A549 and ROSI/H441GL cells but not their respective controls (All experiments were performed in triplicates). Experimental protocol for glucose uptake assay. Cancer cells have been known to exhibit enhanced metabolism, as reflected in the significantly marked glucose uptake ability (Warburg effect). Based on our finding in this study, MKP-1 over-expressing NSCLC cells showed a marked reduction in proliferative ability via ERK-mediated pathway. To further support this notion, we examined the glucose uptake ability of these MKP-1 over-expressing NSCLC cells. Glucose uptake assay was performed in triplicate. NSCLC cells were seeded in 12-well plate at a density of 3 × 10^5^cells/well. Cells were incubated in medium contained 0.1 mM 2-deoxy-D-glucose and 0.5 μCi 2- [1,2-3H]-2-deoxy-D-glucose. Following incubation for 1 h, samples were washed twice with cold Ca2+- and Mg2+-free phosphate-buffer saline. Cells were then lyzed in 10 mM Tris-HCl (pH 8.0) containing 0.2% SDS, the incorporated radioactivity was then determined using TopCount NXT™ Microplate Scintillation and Luminescence Counter (Perkin Elmer, Taipei, Taiwan). [file 1471-2407-10-95-S6.PDF]
